# Supplementary material for: Fructose-1,6-bisphosphate couples glycolytic activity to cell adhesion
Source: Nat Cell Biol. 2026 Mar 16;28(4):739–53. doi: 10.1038/s41556-026-01911-1 (PMC13086585; doi:10.1038/s41556-026-01911-1)
Supplement: Supplementary file 1 — Supplementary Fig. 1 [file 41556_2026_1911_MOESM1_ESM.pdf]

nature cell biology

Supplementary information https://doi.org/10.1038/s41556-026-01911-1

Fructose-1,6-bisphosphate couples glycolytic activity to cell adhesion

In the format provided by the authors and unedited

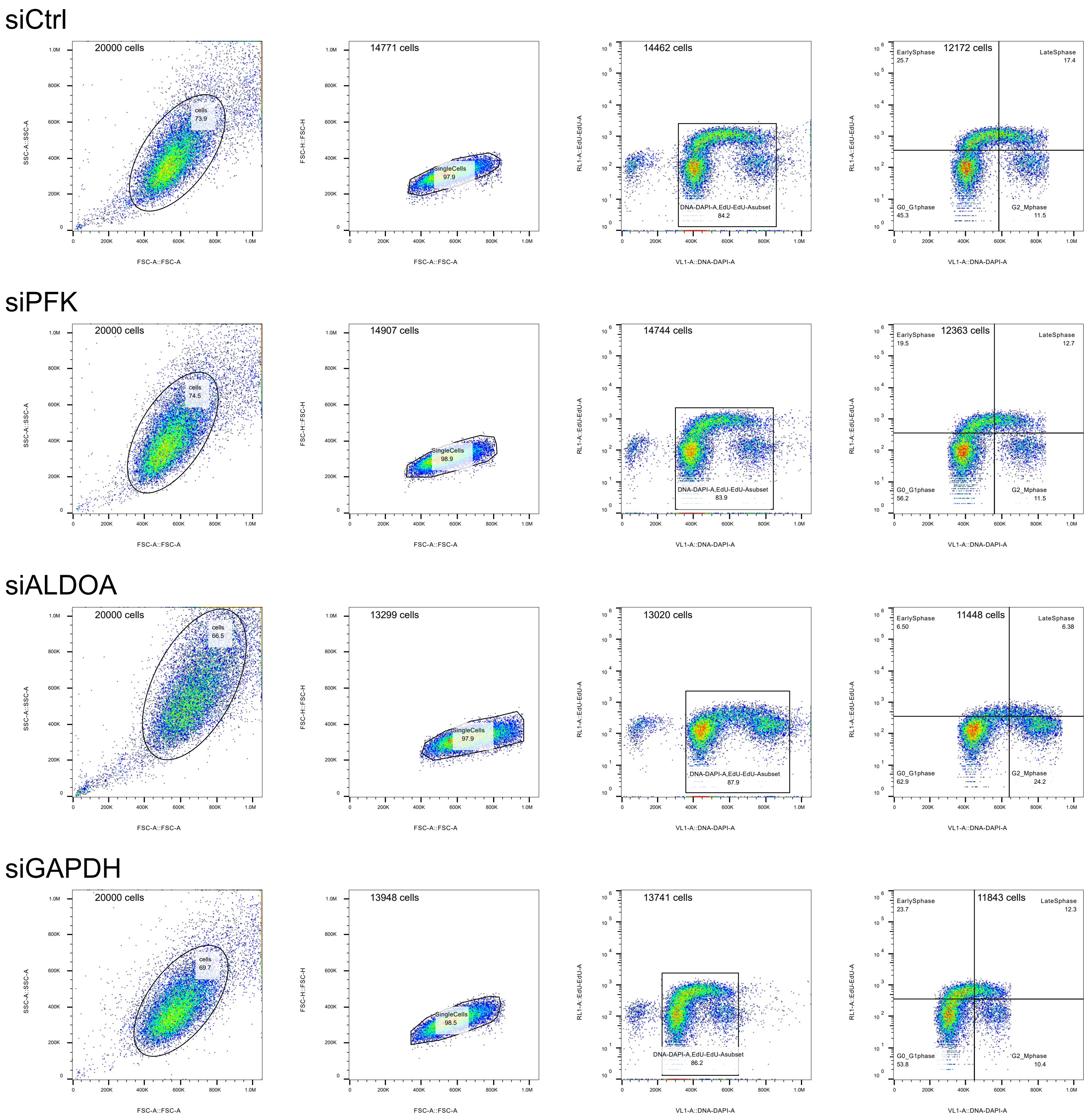

Supplementary Figure 1: Gating strategy and exemplary plots for each condition. First, the actual cell population was defined via the FSC/SSC areas. FSC area vs. FSC height was used to filter single cells. By plotting the DAPI area vs. the EdU area, the fraction of cells in each cell cycle phase (G1/G0, early S phase, late S phase, or G2/M phase) was determined.
