# Supplementary material for: Fructose-1,6-bisphosphate couples glycolytic activity to cell adhesion
Source: Nat Cell Biol. 2026 Mar 16;28(4):739–53. doi: 10.1038/s41556-026-01911-1 (PMC13086585; doi:10.1038/s41556-026-01911-1)
Supplement: Supplementary file 2 — Reporting Summary [file 41556_2026_1911_MOESM2_ESM.pdf]

nature portfolio

Corresponding author(s): Tanja Maritzen

Last updated by author(s): Feb 9, 2026

Reporting Summary

Nature Portfolio wishes to improve the reproducibility of the work that we publish. This form provides structure for consistency and transparency in reporting. For further information on Nature Portfolio policies, see our Editorial Policies and the Editorial Policy Checklist.

Statistics

For all statistical analyses, confirm that the following items are present in the figure legend, table legend, main text, or Methods section.

n/a	Confirmed	
☐	☒	The exact sample size (*n*) for each experimental group/condition, given as a discrete number and unit of measurement
☐	☒	A statement on whether measurements were taken from distinct samples or whether the same sample was measured repeatedly
☐	☒	The statistical test(s) used AND whether they are one- or two-sided<br>*Only common tests should be described solely by name; describe more complex techniques in the Methods section.*
☒	☐	A description of all covariates tested
☐	☒	A description of any assumptions or corrections, such as tests of normality and adjustment for multiple comparisons
☐	☒	A full description of the statistical parameters including central tendency (e.g. means) or other basic estimates (e.g. regression coefficient) AND variation (e.g. standard deviation) or associated estimates of uncertainty (e.g. confidence intervals)
☐	☒	For null hypothesis testing, the test statistic (e.g. *F*, *t*, *r*) with confidence intervals, effect sizes, degrees of freedom and *P* value noted<br>*Give P values as exact values whenever suitable.*
☒	☐	For Bayesian analysis, information on the choice of priors and Markov chain Monte Carlo settings
☒	☐	For hierarchical and complex designs, identification of the appropriate level for tests and full reporting of outcomes
☒	☐	Estimates of effect sizes (e.g. Cohen's *d*, Pearson's *r*), indicating how they were calculated

Our web collection on statistics for biologists contains articles on many of the points above.

Software and code

Policy information about availability of computer code

Data collection	Quantitative values from Western Blots were acquired using the Licor Odyssey software Image Studio Lite (version 5.2 and 6.1). Fluorescence images from Nikon microscopes were acquired with the Nikon NIS Elements software (version 5.21.03). Analysis of microscopy images was performed with KNIME (version 3.7.0), ImageJ/Fiji (version 1.54f), CellProfiler (version 3.1.8) and Python (version 3.8.3). Peptides were modelled using PyMOL (version 3.9). Data were collected in Microsoft Excel files (version 16.104.1).
Data analysis	All statistical tests were performed using GraphPad Prism (version 9 and 10).

For manuscripts utilizing custom algorithms or software that are central to the research but not yet described in published literature, software must be made available to editors and reviewers. We strongly encourage code deposition in a community repository (e.g. GitHub). See the Nature Portfolio guidelines for submitting code & software for further information.

Data

Policy information about availability of data

All manuscripts must include a data availability statement. This statement should provide the following information, where applicable:

- Accession codes, unique identifiers, or web links for publicly available datasets
- A description of any restrictions on data availability
- For clinical datasets or third party data, please ensure that the statement adheres to our policy

Mass spectrometry data have been deposited in ProteomeXchange with the primary accession code PXD043853 [https://www.ebi.ac.uk/pride/archive/projects/

PXD043853]. Data on the genome-wide screen can be found in the Supplementary table. Numerical source data have been provided in Source Data. All other data supporting the findings of this study are available from the corresponding author on reasonable request.

Research involving human participants, their data, or biological material

Policy information about studies with human participants or human data. See also policy information about sex, gender (identity/presentation), and sexual orientation and race, ethnicity and racism.

Reporting on sex and gender	Sex and gender were not considered in our study design since our study is based on human cell lines and investigates basic cellular processes that are regarded as independent of sex and gender. The sex of the used cell lines was reported as far as it was known.
Reporting on race, ethnicity, or other socially relevant groupings	We do not use socially constructed or socially relevant categorization variables in our study.
Population characteristics	Our study does not involve human research participants.
Recruitment	Our study did not involve the recruitment of participants.
Ethics oversight	The study protocol did not need to be approved by an ethics committee using exclusively standard cell lines.

Note that full information on the approval of the study protocol must also be provided in the manuscript.

Field-specific reporting

Please select the one below that is the best fit for your research. If you are not sure, read the appropriate sections before making your selection.

☒ Life sciences ☐ Behavioural & social sciences ☐ Ecological, evolutionary & environmental sciences

For a reference copy of the document with all sections, see nature.com/documents/nr-reporting-summary-flat.pdf

Life sciences study design

All studies must disclose on these points even when the disclosure is negative.

Sample size	Sample sizes were not chosen based on pre-specified effect size, as the effect sizes were not known beforehand, but had to be discovered as part of the study. Instead, a sufficient number of independent experiments was carried out (as detailed in the figure legends) to allow for statistical testing.
Data exclusions	No samples were excluded from analysis.
Replication	Experiments were replicated independently. All attempts of replication were successful.
Randomization	Cell culture dishes were allocated in a randomized manner to treatment conditions.
Blinding	The genome-wide screen with its automated analysis pipeline was objective by design. For subsequent experiments, blinding during the experiment was often not applicable as most knockdowns (PFK, Aldolase, RCC2, Rac1) caused obvious morphological changes in the cells. To avoid potential bias in imaging experiments, cells for analysis were chosen based solely on their nuclear staining and image analysis was performed in an automated fashion. Cells for Western blots were not collected or processed blindly since knowledge of the characteristic of each sample was necessary for data generation.

Reporting for specific materials, systems and methods

We require information from authors about some types of materials, experimental systems and methods used in many studies. Here, indicate whether each material, system or method listed is relevant to your study. If you are not sure if a list item applies to your research, read the appropriate section before selecting a response.

Materials & experimental systems

n/a	Involved in the study
☐	☒ Antibodies
☐	☒ Eukaryotic cell lines
☒	☐ Palaeontology and archaeology
☒	☐ Animals and other organisms
☒	☐ Clinical data
☒	☐ Dual use research of concern
☒	☐ Plants

Methods

n/a	Involved in the study
☒	☐ ChIP-seq
☐	☒ Flow cytometry
☒	☐ MRI-based neuroimaging

Antibodies

Antibodies used

A complete list of the antibodies used in this study can be found in the Supplementary Table and under the following point.

Validation

Paxillin (Abcam, ab32084): specificity was validated by knockdown in this study, see Extended Fig. 1c; Aldolase (Proteintech, 11217-1-AP): specificity was validated by knockdown in this study, see Fig. 2e, Fig. 3b, Fig. 5e, Fig. 6c, Fig. 6d, etc.; PFKM (Proteintech, 55028-1-AP): specificity was validated by knockdown in this study, see Fig. 2e, Fig. 3b; PFKL (Santa Cruz, sc-393713): specificity was validated by knockdown in this study, see Fig. 2e, Fig. 3b; PFKP (Cell Signaling, 8164): specificity was validated by knockdown in this study, Fig. 2e, see Fig. 3b; GAPDH (Merck, G8795): specificity was validated by knockdown in this study, see Fig. 2e, Extended Fig. 3a, e, i; Rac1 (BD Transduction, 610650): specificity was validated by knockdown in this study, see Fig. 6d, Fig. 8h; RCC2 (Novus Biological, NB110-40618): specificity was validated by knockdown in this study, see Fig. 8d, Fig. 8h; Clathrin heavy chain (Abcam, ab21679): specificity was validated by knockdown in https://doi.org/10.1371/journal.pone.0003115; Alpha-tubulin (Merck, T5168): specificity was demonstrated by immunofluorescence and Western blot using lysates from various cell lines according to the manufacturer's website (https://www.sigmaaldrich.com/DE/de/product/sigma/t5168); beta-actin (Merck, A5441): specificity was validated by knockdown in https://doi.org/10.1091/mbc.e11-06-0582; c-Myc (Merck, M4439): specificity was demonstrated by Western blot and ChiP according to manufacturer's website (https://www.sigmaaldrich.com/DE/de/product/sigma/m4439); GFP (Abcam, ab13970): Specificity was demonstated by Western blot and Immunofluorescence after overexpression according to manufacturer's website (https://www.abcam.com/en-us/products/primary-antibodies/gfp-antibody-ab13970) [see also Supplementary Table 4]

Eukaryotic cell lines

Policy information about cell lines and Sex and Gender in Research

Cell line source(s)

The following cell lines were obtained from the American Type Culture Collection (ATCC): the human sarcoma cell line U-2 OS (ATCC: HTB-96; female), the human embryonic kidney cell line HEK293T (ATCC: CRL-3216; sex not specified), the human breast cancer cell line MDA-MB-231 (ATCC: HTB-26; female), and the telomerase-immortalized human retinal pigment epithelium cell line hTERT RPE-1 (ATTC: CRL-4000; female). Telomerase-immortalized human foreskin fibroblasts (HFF; sex not specified) were a kind gift of Prof. Martin J. Humphries (University of Manchester, UK).

Authentication

The cell lines were authenticated by the vendor ATCC by short tandem repeat (STR) marker profiling . The only non-commercial cell line used (HFF) which we obtained from another lab was authenticated based on their typical fibroblast morphology.

Mycoplasma contamination

Cell lines were regularly tested for mycoplasma contamination and were not contaminated.

Commonly misidentified lines
(See ICLAC register)

N/A

Flow Cytometry

Plots

Confirm that:

☒ The axis labels state the marker and fluorochrome used (e.g. CD4-FITC).

☒ The axis scales are clearly visible. Include numbers along axes only for bottom left plot of group (a 'group' is an analysis of identical markers).

☒ All plots are contour plots with outliers or pseudocolor plots.

☒ A numerical value for number of cells or percentage (with statistics) is provided.

Methodology

Sample preparation

1.5 million U-2 OS cells were seeded into 10 cm dishes. On the next day, their cell cycle was analyzed via the following steps where all centrifugation steps were performed at room temperature and 400 g for 5 min. 10 µM EdU (Merck) were added to the cells for 30 min at 37°C to label the cells in S Phase. After trypsination, the cells were washed in PBS, centrifuged, and

fixed and permeabilized in Fixation/Permeabilization solution (1:3 Fixation/Permeabilization Concentrate in Fixation/Permeabilization Dilution, InvitrogenTM) at room temperature for 15 min. 1 ml PBS was added, and after centrifugation the cell pellet was resuspended in 100 µl Perm Wash (1:10 Permeabilization Buffer (InvitrogenTM) in H2O). EdU-containing cells were fluorescently labeled with Eterneon-Red (baseclick GmbH) via incubation in the ClickIt Reaction Mix (1 µM Eterneon-Red, 10 mM Tris pH 8.8, 500 µM CuSO4, and 100 mM Ascorbic Acid, in PBS) for 20 min. After two washes in Perm Wash, the cells were stained with 0.2 µg/ml DAPI in PBS for at least 5 min. Eterneon-Red and DAPI intensities were measured via flow cytometry.

Instrument

Attune™ NxT Flow Cytometer (InvitrogenTM)

Software

FlowJoTM v10.8 Software (BD Life Sciences)

Cell population abundance

20000 cells were measured per condition. The supplementary figure exemplifying the gating strategy also contains information on the abundance of the relevant cell populations within the sorted fractions.

Gating strategy

The cell population was first defined via the forward and side scatter pulse areas. Via gating of the forward scatter area and forward scatter height, doublets were excluded. After plotting the area of the DAPI signal vs. the area of the EdU signal, the main cell cluster was defined as the final subset of cells. Cells with an Eterneon-Red pulse area above 350 were classified as cells in S phase. Cells with a pulse area below 350 were further categorized as cells in either G0/G1 phase (low DAPI pulse area) or G2/M phase (high DAPI pulse area), as follows: Borders were manually defined around the two visible subpopulations as exemplified in the supplementary figure on the gating strategy. The center between the two inner borders was set as the threshold between low and high DAPI pulse areas.

☒ Tick this box to confirm that a figure exemplifying the gating strategy is provided in the Supplementary Information.
