# Supplementary material for: Fructose-1,6-bisphosphate couples glycolytic activity to cell adhesion
Source: Nat Cell Biol. 2026 Mar 16;28(4):739–53. doi: 10.1038/s41556-026-01911-1 (PMC13086585; doi:10.1038/s41556-026-01911-1)

Fig. 2e

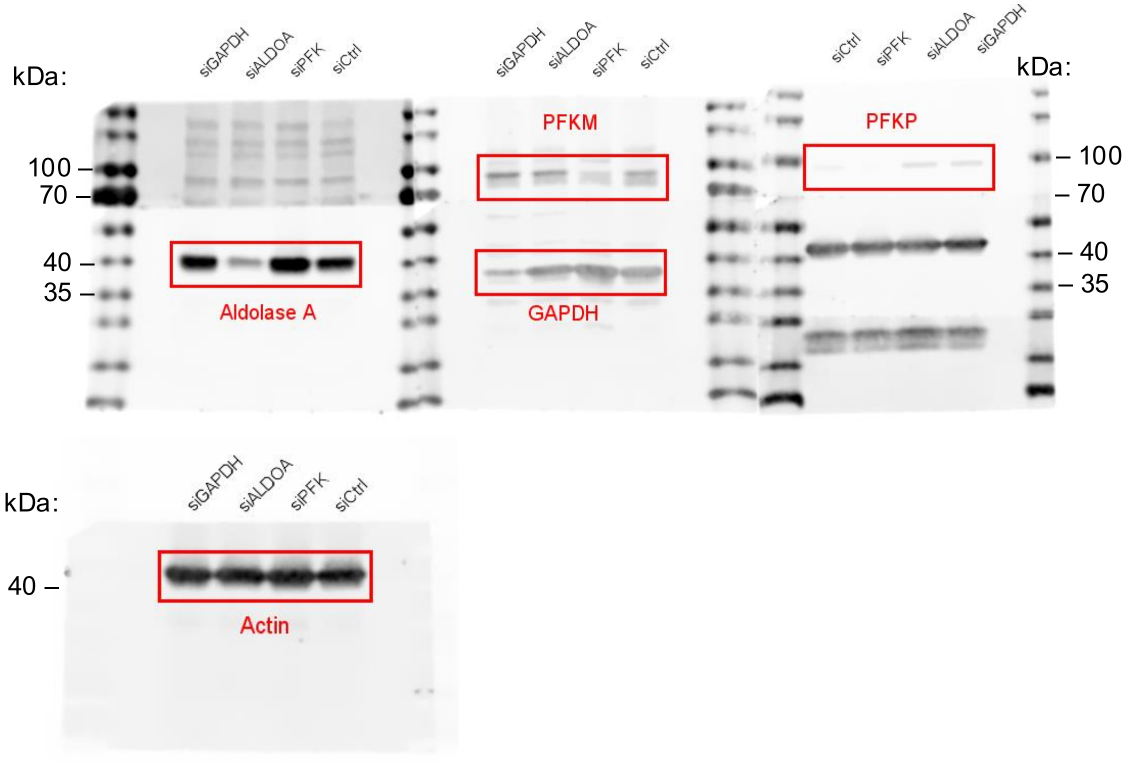

kDa:
siGAPDH
siALDOA
siPFK
siCtrl
100
70
40
35
Aldolase A
siGAPDH
siALDOA
siPFK
siCtrl
PFKM
GAPDH
siCtrl
siPFK
siALDOA
siGAPDH
kDa:
PFKP
100
70
40
35
kDa:
siGAPDH
siALDOA
siPFK
siCtrl
40
Actin

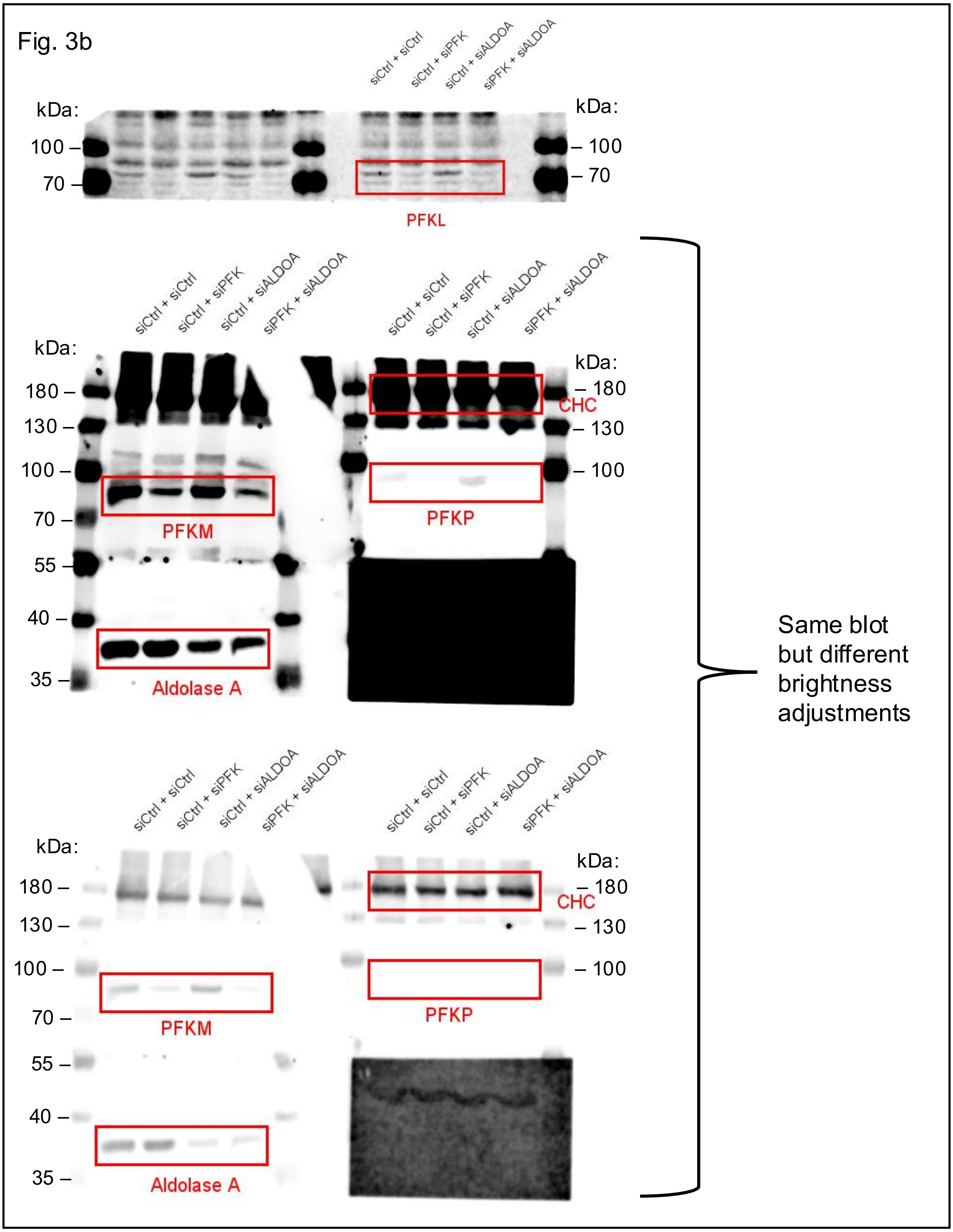

Fig. 3b
siCtrl + siCtrl
siCtrl + siPFK
siCtrl + siALDOA
siPFK + siALDOA
kDa:
100
70
PFKL
PFKM
Aldolase A
PFKP
CHC
180
130
55
40
35
Same blot but different brightness adjustments

Fig. 5e

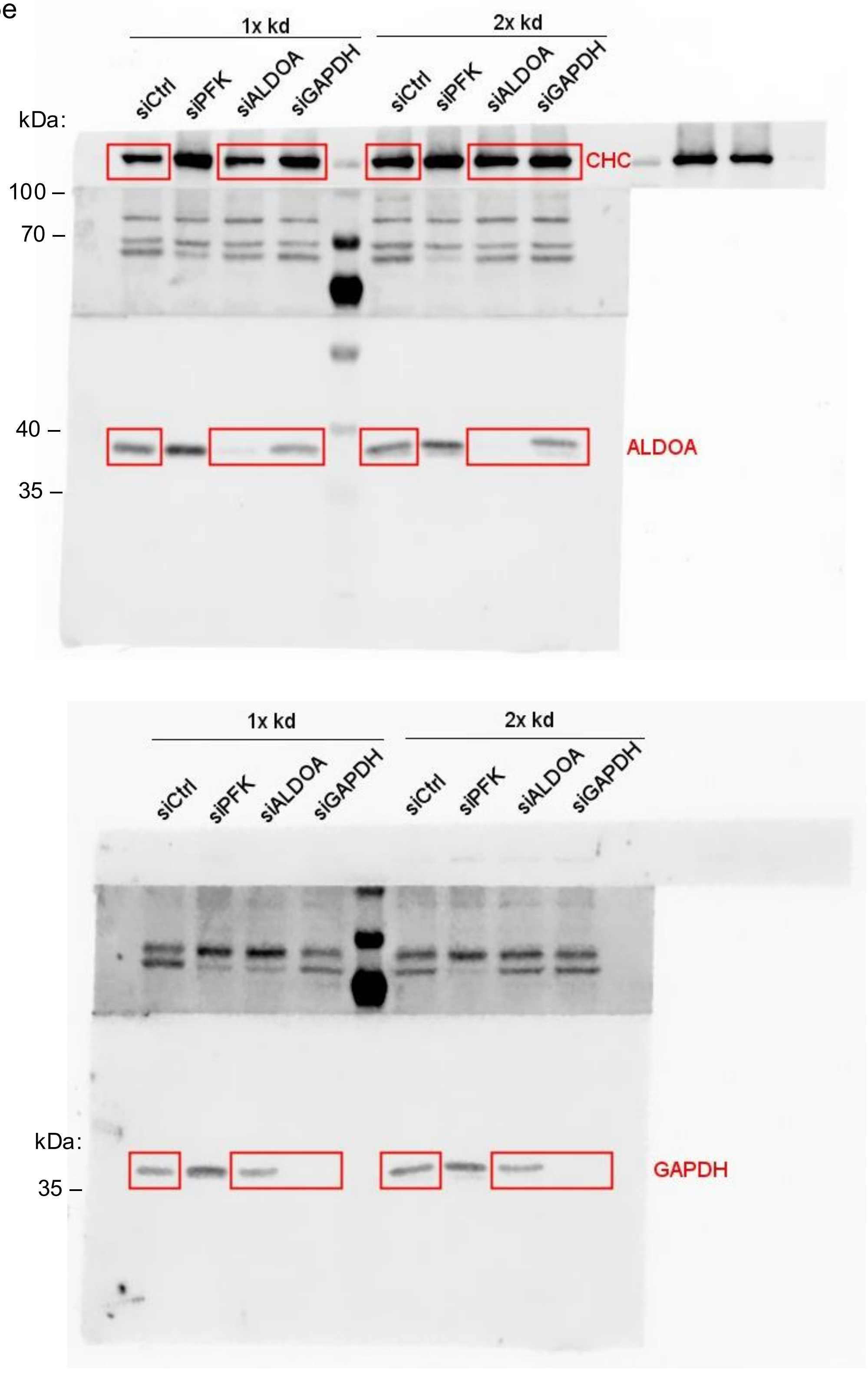

1x kd
2x kd
siCtrl
siPFK
siALDOA
siGAPDH
siCtrl
siPFK
siALDOA
siGAPDH
kDa:
100 –
70 –
40 –
35 –
CHC
ALDOA
1x kd
2x kd
siCtrl
siPFK
siALDOA
siGAPDH
siCtrl
siPFK
siALDOA
siGAPDH
kDa:
35 –
GAPDH

Fig. 6c

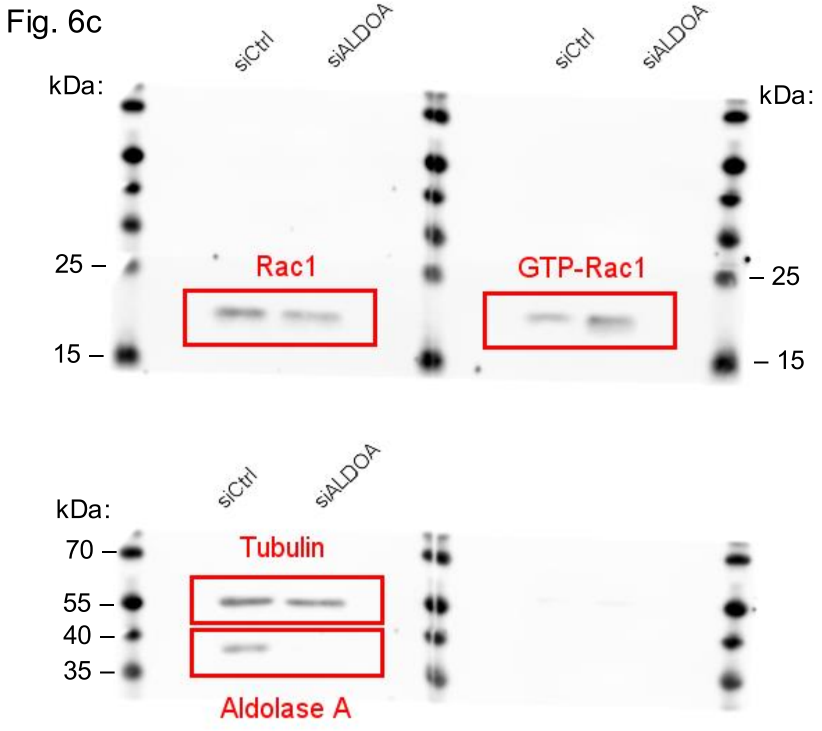
siCtrl
siALDOA
siCtrl
siALDOA
kDa:
kDa:
25
15
Rac1
GTP-Rac1
25
15
siCtrl
siALDOA
kDa:
70
55
40
35
Tubulin
Aldolase A

Fig. 6d

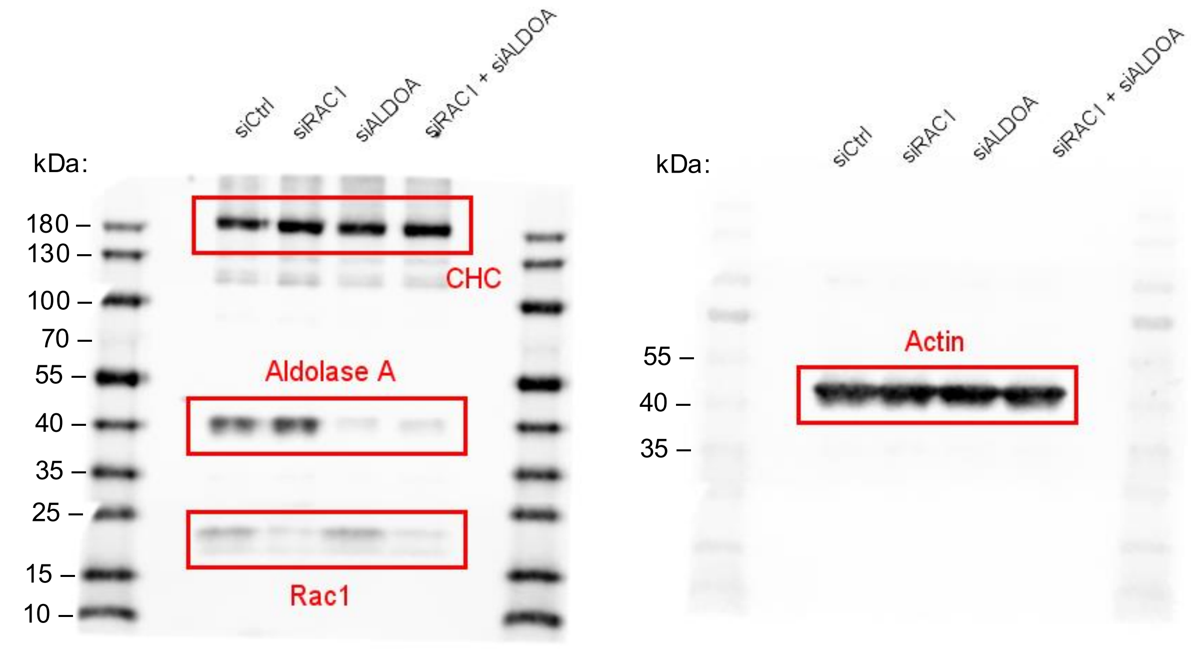
siCtrl
siRAC1
siALDOA
siRAC1 + siALDOA
kDa:
180
130
100
70
55
40
35
25
15
10
CHC
Aldolase A
Rac1
siCtrl
siRAC1
siALDOA
siRAC1 + siALDOA
kDa:
55
40
35
Actin

Fig. 7g

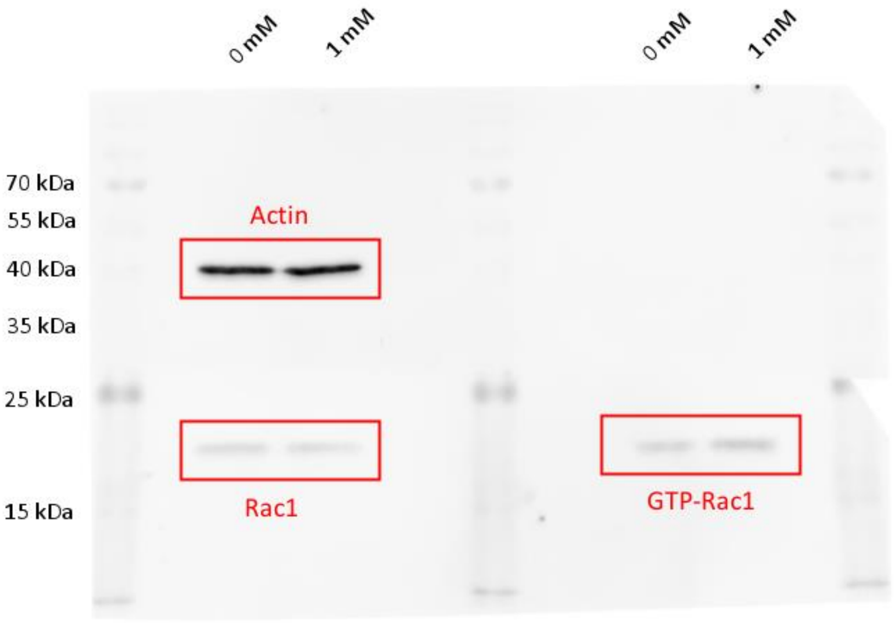
0 mM
1 mM
0 mM
1 mM
70 kDa
55 kDa
40 kDa
35 kDa
25 kDa
15 kDa
Actin
Rac1
GTP-Rac1

Fig. 8d

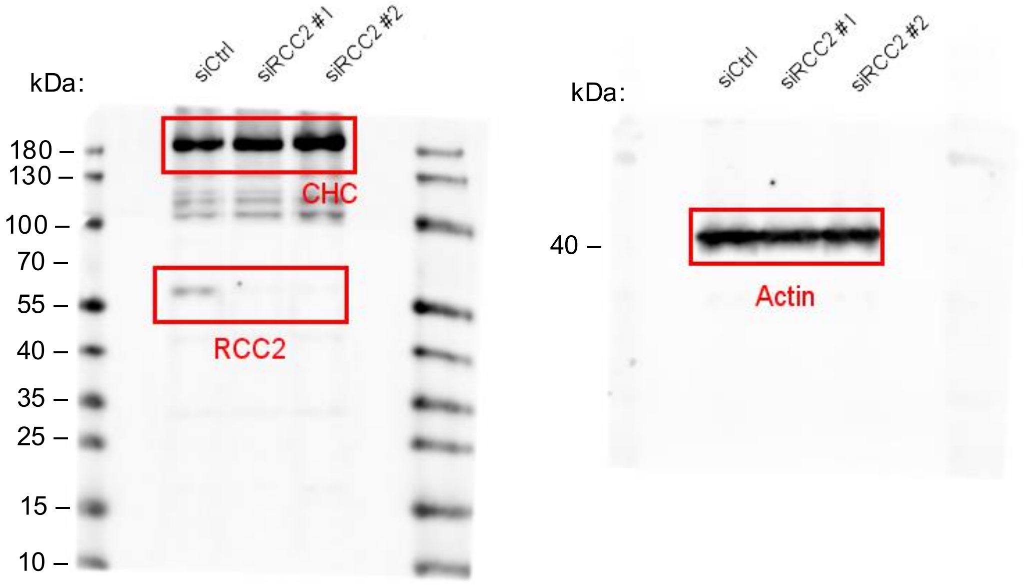

siCtrl
siRCC2 #1
siRCC2 #2
kDa:
180 –
130 –
100 –
70 –
55 –
40 –
35 –
25 –
15 –
10 –
CHC
RCC2
siCtrl
siRCC2 #1
siRCC2 #2
kDa:
40 –
Actin


Fig. 8h

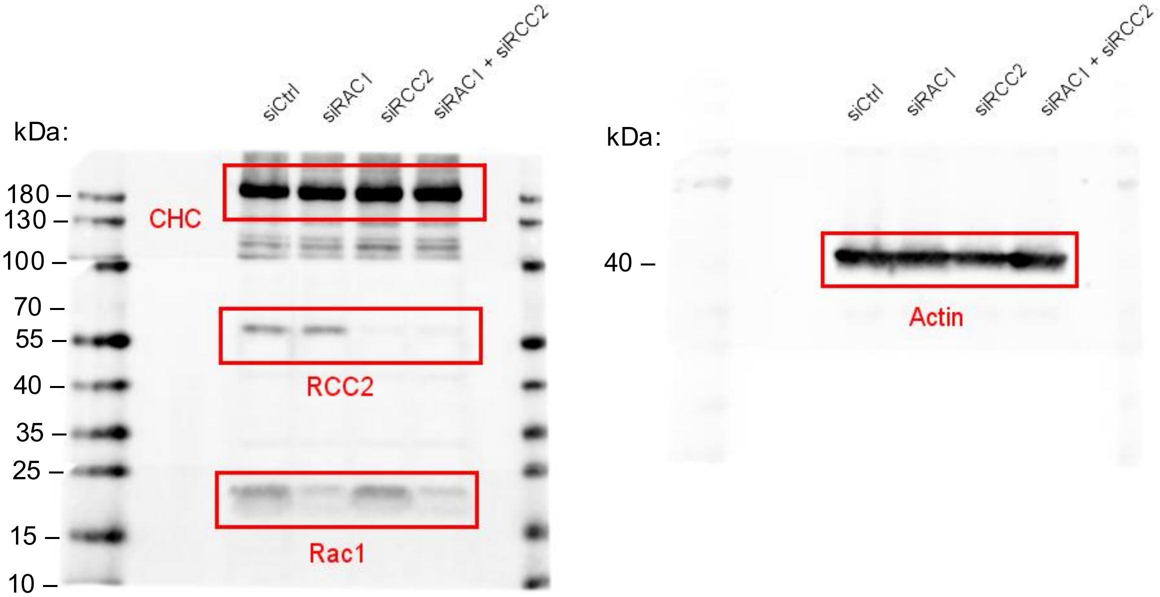

siCtrl
siRAC1
siRCC2
siRAC1 + siRCC2
kDa:
180 –
130 –
100 –
70 –
55 –
40 –
35 –
25 –
15 –
10 –
CHC
RCC2
Rac1
siCtrl
siRAC1
siRCC2
siRAC1 + siRCC2
kDa:
40 –
Actin

Fig. 8m

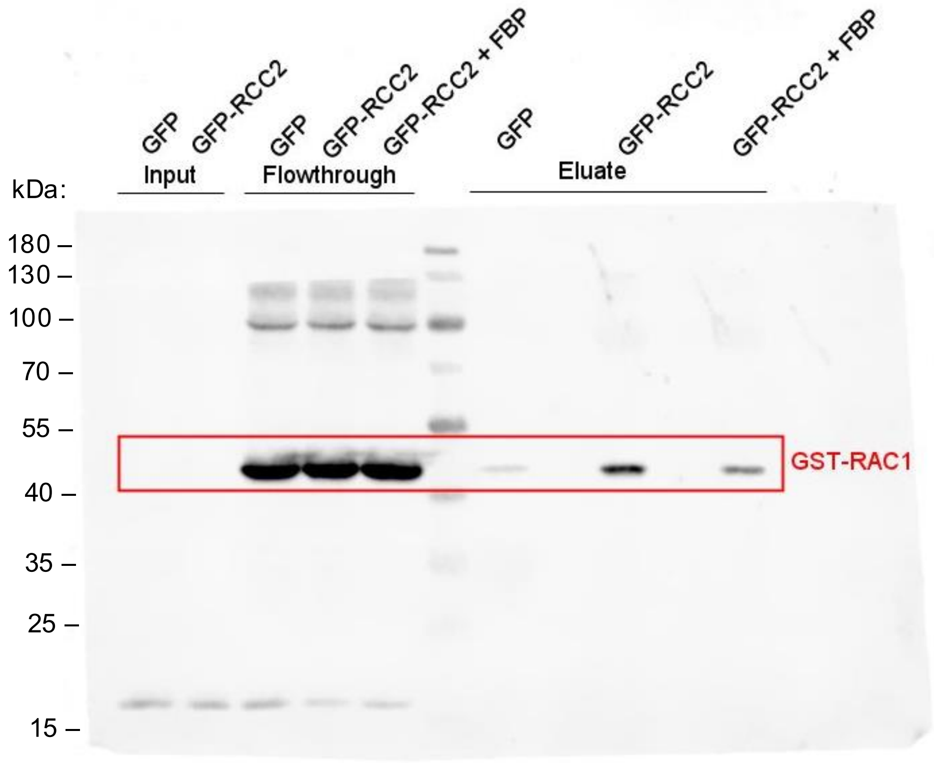
GFP
GFP-RCC2
GFP
GFP-RCC2
GFP-RCC2 + FBP
GFP
GFP-RCC2
GFP-RCC2 + FBP
Input
Flowthrough
Eluate
kDa:
180 –
130 –
100 –
70 –
55 –
40 –
35 –
25 –
15 –
GST-RAC1

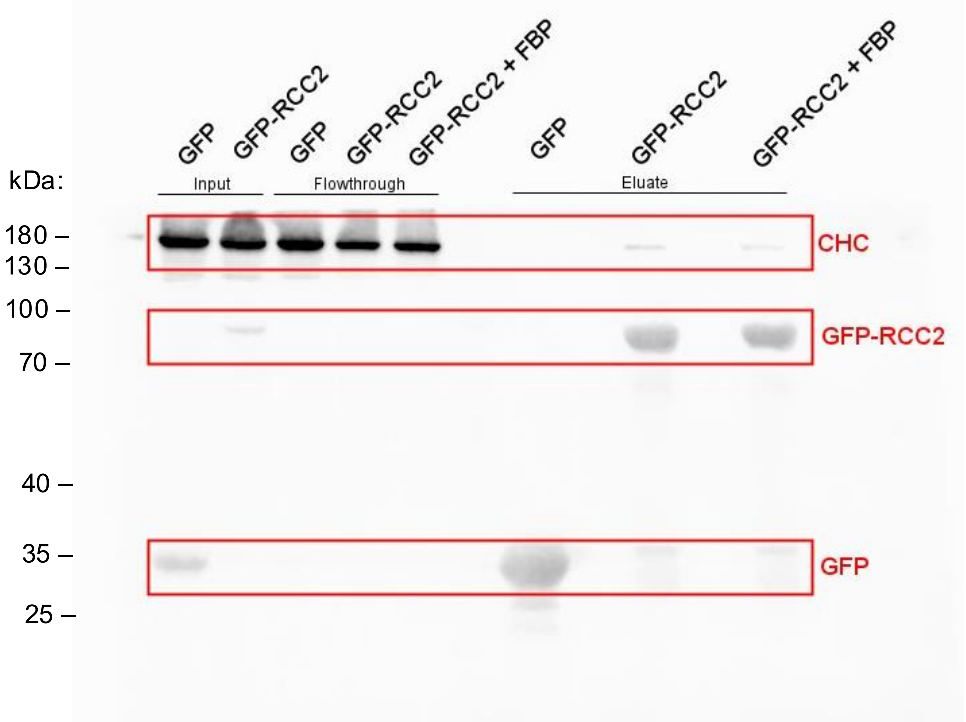
GFP
GFP-RCC2
GFP
GFP-RCC2
GFP-RCC2 + FBP
GFP
GFP-RCC2
GFP-RCC2 + FBP
kDa:
Input
Flowthrough
Eluate
180 –
130 –
100 –
70 –
40 –
35 –
25 –
CHC
GFP-RCC2
GFP

Extended Data Fig. 2a

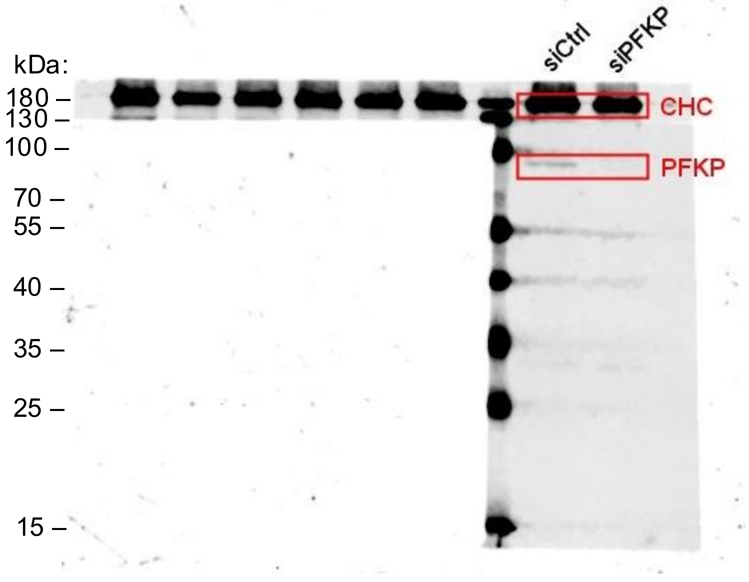

## Extended Data Fig. 3a

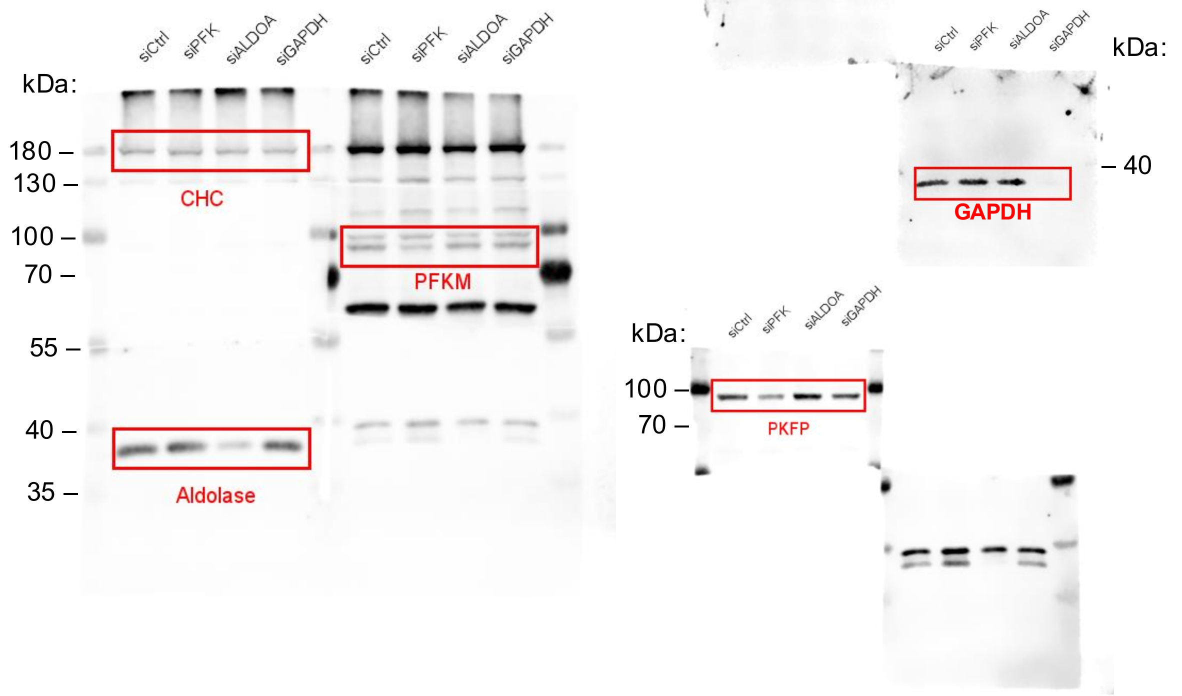


## Extended Data Fig. 3e

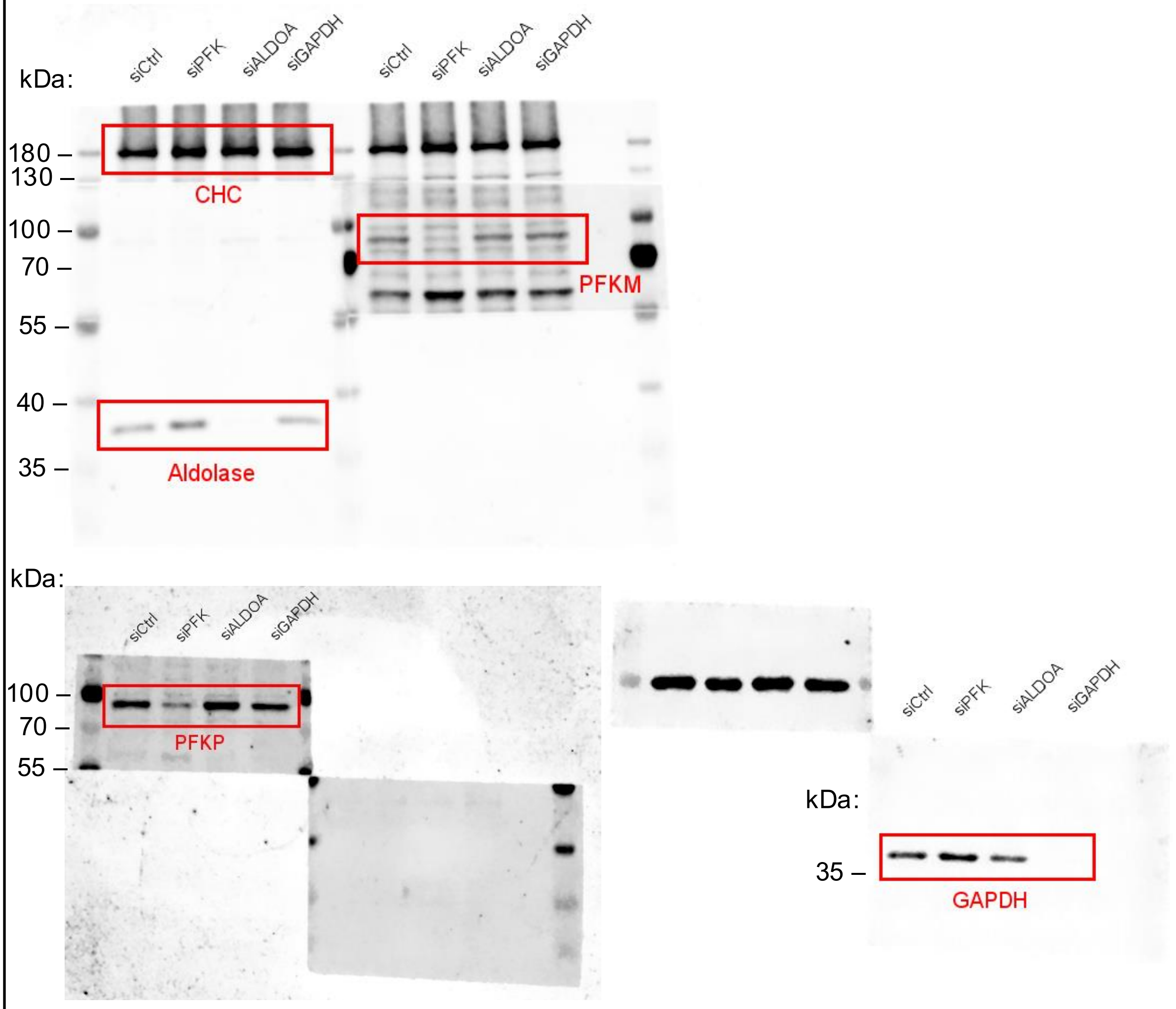

Extended Data Fig. 3i

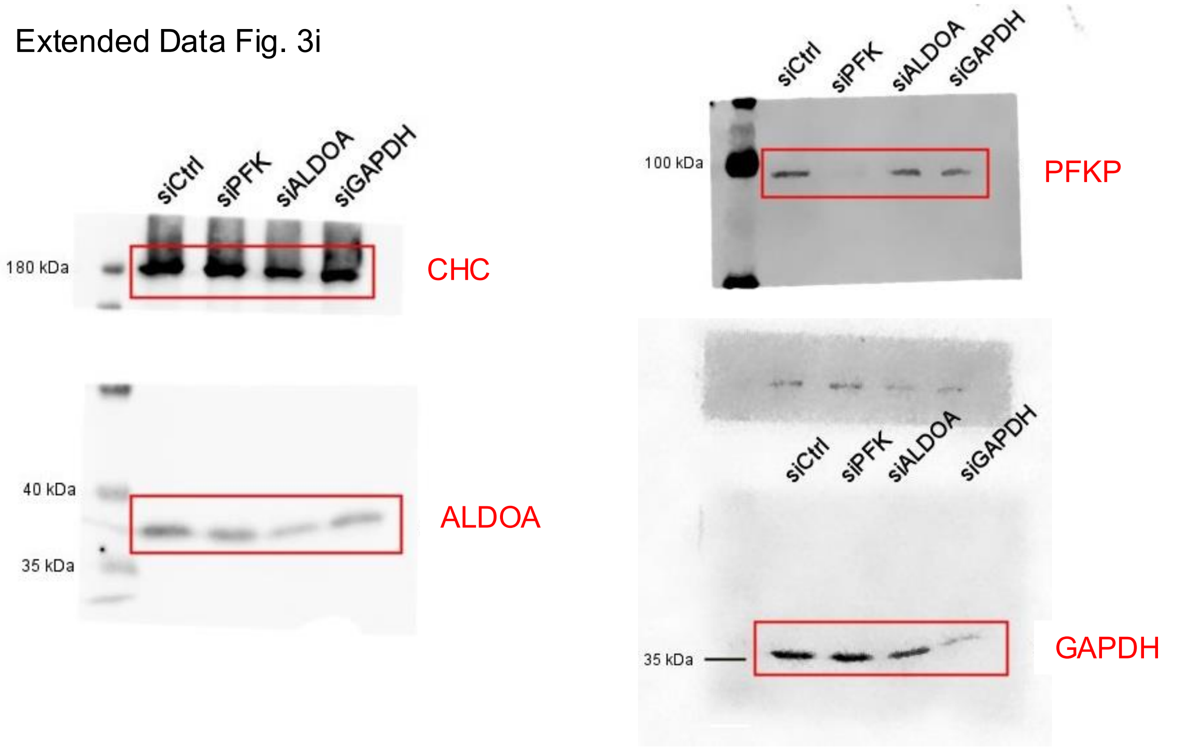

Extended Data Fig. 7a

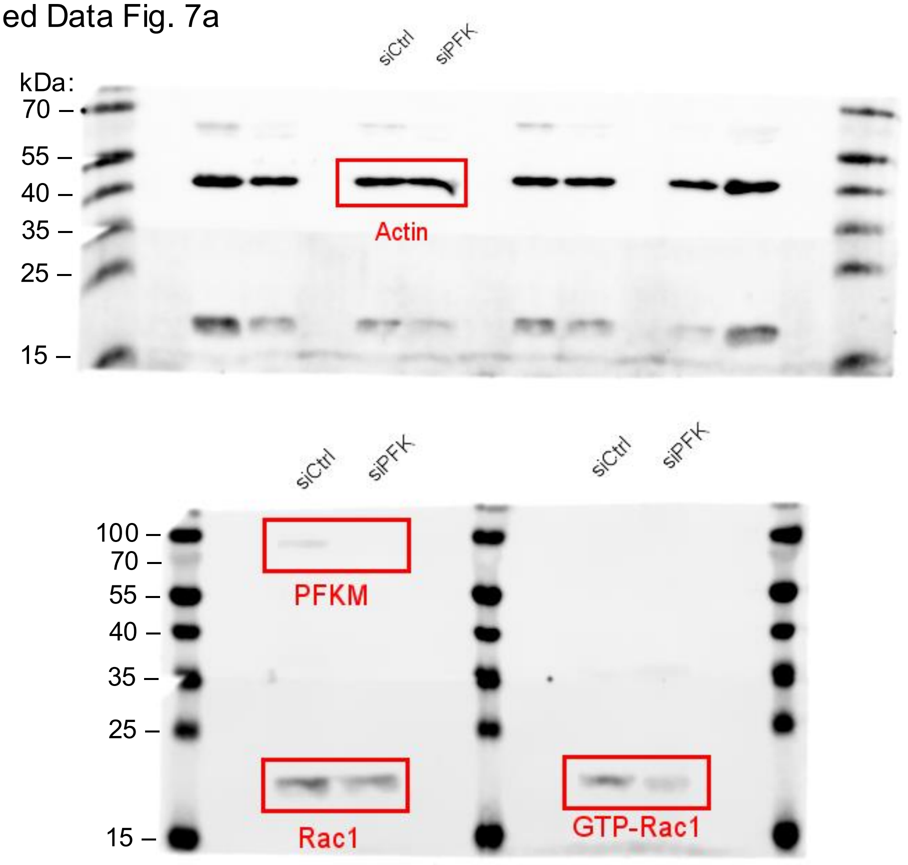


Extended Data Fig. 7e

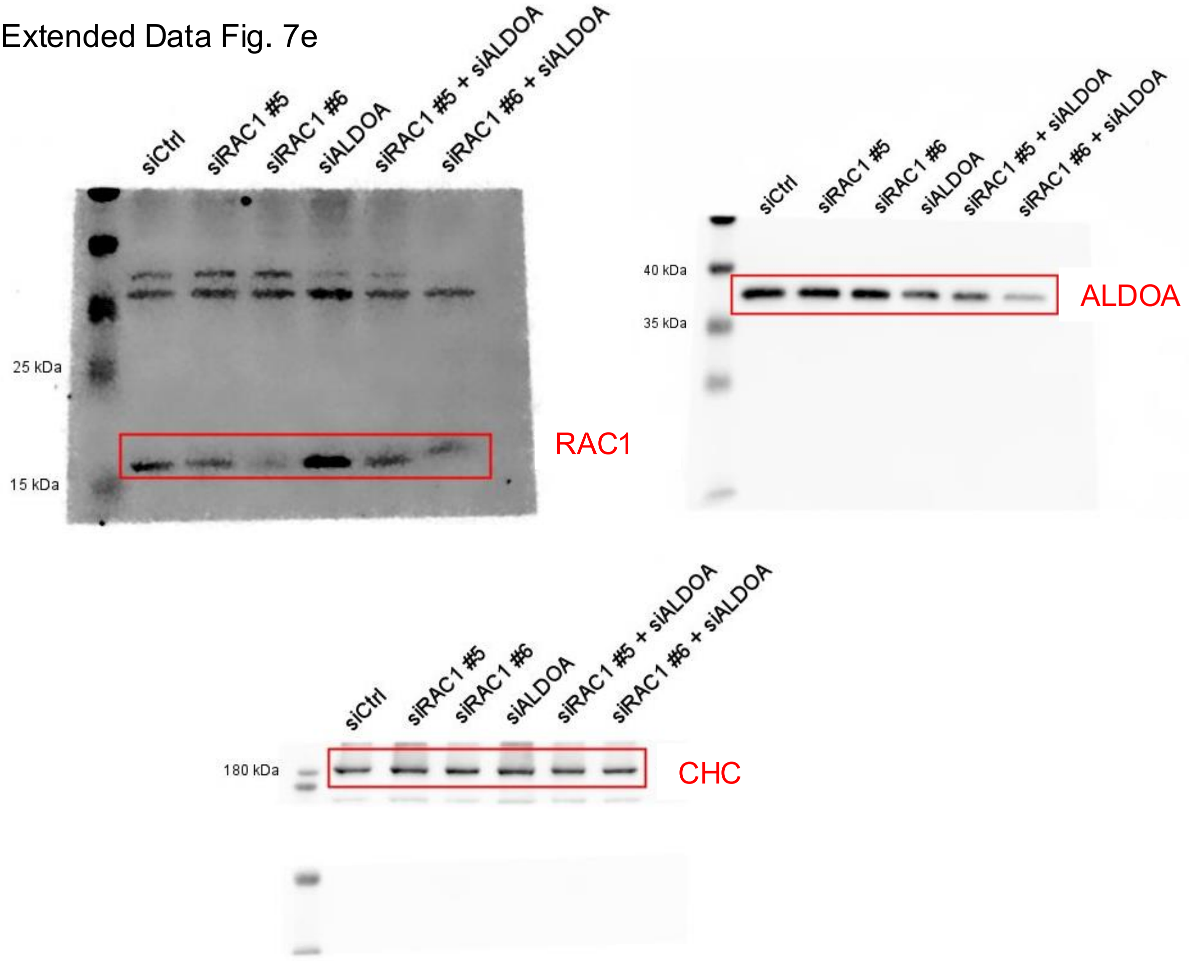

Supplement: Supplementary file 10 — Compilation of unprocessed versions of all western blot images displayed in Figs. 1–8 and Extended Data Figs. 1–9. [file 41556_2026_1911_MOESM10_ESM.pdf]
